# Marbelization of the gallbladder, a variant of acute gangrenous cholecystitis

**DOI:** 10.4314/gmj.v55i3.8

**Published:** 2021-09

**Authors:** Ismail Burud, Mahadevan D Tata, Kogaan Selvaraja, Sherreen Y El Hariri

**Affiliations:** 1 Department of Surgery, International Medical University, Clinical campus, Seremban, 70300, Negeri Sembilan, Malaysia; 2 Department of Surgery, CMH Specialist Hospital, Seremban, 70200, Negeri Sembilan, Malaysia

**Keywords:** gangrenous cholecystitis, emphysematous cholecystitis, marbleization of the gallbladder, acute cholecystitis

## Abstract

Cholelithiasis can present from a milder form of biliary colic to a more severe and complicated one like empyema gallbladder and a lesser-known variant of gangrenous gallbladder called marbleization of the gallbladder. The clinical signs and symptoms are similar to acute cholecystitis. Diabetes mellitus could have a role in the process of marbleization. Diagnosing marbleization of the gall bladder is not easy preoperatively. Computerized tomography is a better diagnostic modality when compared to laboratory investigations. Urgent cholecystectomy is the only option, and there is no role of conservative treatment. We report a case of a 36-year-old man with newly diagnosed Diabetes Mellitus diagnosed initially as acute cholecystitis and managed conservatively. He did not respond to treatment and hence underwent cholecystectomy and intraoperatively was found to have marbleization of the gall bladder.

## Introduction

Cholelithiasis and its related complications are commonly seen in the surgical wards. The various complications that Cholelithiasis can cause are acute cholecystitis, ascending cholangitis, biliary pancreatitis, etc.^1^ It can develop into rare complications such as empyema gall bladder and gangrenous gall bladder that are life-threatening. There is another variant of gangrenous cholecystitis (GC) that many are unaware where coalescing of the bile sludge with necrosis, withered in the outer layer of gall bladder giving an appearance of shiny dark green marblelike appearance. This is called the marbleization of the gall bladder (MGB). This eventually will cause perforation of the gall bladder. It is an intraoperative finding, and when seen, cholecystectomy is the only option. We would like to report a rare occurrence of untreated or partially treated acute cholecystitis, the marbleized gall bladder.

## Case Report

A 36-year-old man with newly diagnosed Diabetes Mellitus presented with acute right hypochondriac pain associated with colicky abdominal pain for one week. He started to vomit on the day of admission. The patient was admitted to General Hospital and was treated as acute cholecystitis for four days before he came to our Centre. He did not have a fever or jaundice

On examination, his vital signs were normal. He was not anaemic or jaundiced, and oxygen saturation was 99%. His upper abdomen was guarded with marked tenderness over the right hypochondriac region and murphy sign positive. His Erythrocyte Sedimentation Rate: 127 mm/hr., WBC: 14.1×10^9 /L, Blood Glucose:15.1 mmol/l, Alkaline phosphatase: 218 IU/L

CT abdomen showed distended gallbladder with hyperdense layering seen at the dependent part with pericholecystic fluid collection and there was a breach of gallbladder wall at the posterior aspect. The adjacent duodenum (D2) was compressed with gallbladder fluid. No intrahepatic or extrahepatic duct dilatation was noted.

**Figure 1:**
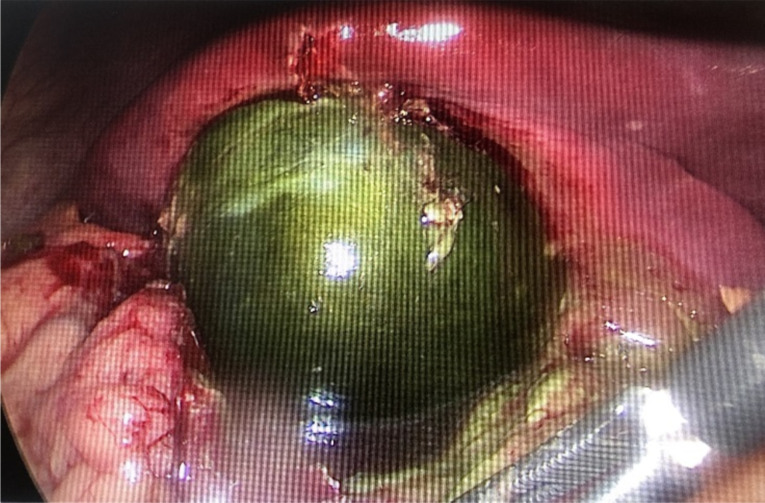
Marbleization of the gall Bladder

**Figure 2:**
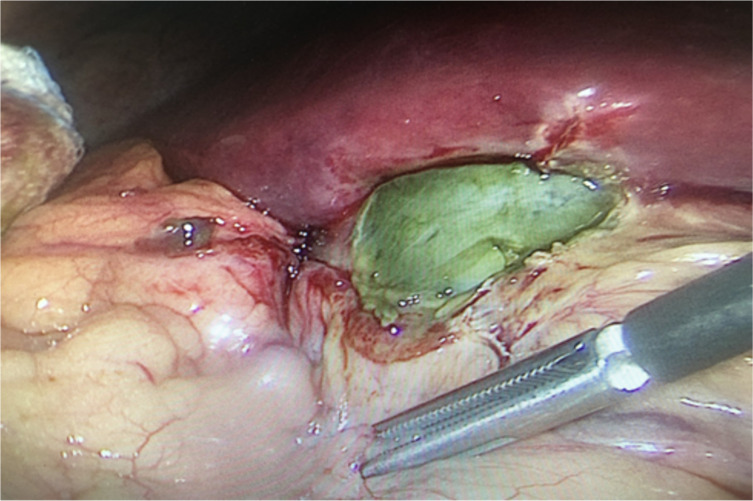
Adhesions Surrounding the gall bladder

Laparoscopic cholecystectomy was done the next day. Intraoperatively there was omental and peritoneal adhesion which were released. A shiny dark green marble-like structure was identified as a gall bladder (Figure 1). The gall bladder was very soft, contained pus, and its wall was necrosed with multiple adhesions (Figure 2). Surrounding adhesion was released, hemostasis secured with careful gall bladder dissection with peritoneal lavage was done.

Gall Bladder was removed without any complication. The patient was well post-operatively and kept on antibiotics for five days. He was discharged uneventfully.

## Discussion

The incidence of GC ranges from 2%to 30% in all patients with acute cholecystitis.^2^ Risk factors for developing GC are male gender, advanced age, delayed surgery, leukocytosis, and comorbidities like cardiovascular diseases and diabetes mellitus. Various factors that predict a tendency towards GC are age, gender, gall bladder wall thickness, white blood cell count, and heart rate.^3^

If left untreated, acute cholecystitis may progress to emphysematous gall bladder, gangrenous gall bladder, or perforated gall bladder. It is important to diagnose complicated acute cholecystitis, mainly GC, correctly and early as it is associated with increased morbidity and mortality.^4^,^5^ In GC, the increased pressure and distension in the gallbladder causes ischemia and necrosis of the gall bladder, which eventually leads to perforation.^6^

Marbleization of tissues is commonly encountered in forensic medicine where putrefaction occurs. After death, the organisms enter the blood vessels and use blood as the culture media, causing tissue disintegration and bacterial decomposition giving rise to a greenish external appearance to the tissues called marbleization.^7^ The patient intraoperatively had marbleization of the gallbladder. In marbleized gall bladder, the mucosal, lamina propria, and smooth muscle layers are partly necrosed or disintegrated. Thus, the bile seeped between the muscular and serosal layers and lay contained within the visceral peritoneum. Bacterial contamination and inflammation of the wall and ischemia result in a bright green marble-like appearing gall bladder that is soft and brittle to touch. This variant of GC needs special attention during treatment and resection to prevent bile spillage and related complications during surgery. There could be a role of diabetes mellitus in the process of marbleization as evident in studies conducted on rats showed that when Free Fatty Acids rose to levels higher than >1.5 mmol/1 and “marbleization” of tissues occurred as Fatty Acyl CoA increasingly esterified to triglycerides.^8^ Our Patient was newly diagnosed to have diabetes.

Diagnosing MGB can be difficult due to the subtle symptoms masked by existing comorbidities. Imaging modality can assist in diagnosing MGB rather than laboratory tests and Computerized Tomography (CT) has a better and accurate result. Important findings on CT are lack of mural enhancement with gallbladder distension, thickening of the wall, and pericholecystic fluid collection.^9^ CT of the patient showed gall bladder distension with hypodense layering in the dependent area. The pericholecystic fluid is seen with a breach of the gall bladder wall at the posterior aspect.

Our patient presented with minimal symptoms and was afebrile which could be due to the concealed perforation of the gall bladder as it was contained due to the omental covering. He was newly diagnosed with Type 2 Diabetes Mellitus. Diabetes mellitus can cause an immunocompromised state. The symptoms were also minimal due to the effect of DM on the immune system. Prior to admission to our Centre, he had been treated partially with antibiotics. This has reduced the severity of the gangrenous gall bladder. The ideal treatment for GC has been proposed to be open or laparoscopic cholecystectomy by many authors.

## Conclusion

MGB is an intraoperative finding difficult to diagnose preoperatively as clinical laboratory and imaging modalities are nonconclusive. Early laparoscopic or open cholecystectomy with careful dissection of the GB and good peritoneal lavage can improve the patient's outcome.
